# Chick embryo xenograft model reveals a novel perineural niche for human adipose-derived stromal cells

**DOI:** 10.1242/bio.010256

**Published:** 2015-08-28

**Authors:** Ingrid R. Cordeiro, Daiana V. Lopes, José G. Abreu, Katia Carneiro, Maria I. D. Rossi, José M. Brito

**Affiliations:** Morphological Sciences Program, Biomedical Sciences Institute, Federal University of Rio de Janeiro, Rio de Janeiro 21941-901, Brazil

**Keywords:** Adipose-derived stromal cell, *Alu*, Chick embryo, Niche, Stem cell, Xenograft

## Abstract

Human adipose-derived stromal cells (hADSC) are a heterogeneous cell population that contains adult multipotent stem cells. Although it is well established that hADSC have skeletal potential *in vivo* in adult organisms, *in vitro* assays suggest further differentiation capacity, such as into glia. Thus, we propose that grafting hADSC into the embryo can provide them with a much more instructive microenvironment, allowing the human cells to adopt diverse fates or niches. Here, hADSC spheroids were grafted into either the presumptive presomitic mesoderm or the first branchial arch (BA1) regions of chick embryos. Cells were identified without previous manipulations via human-specific *Alu* probes, which allows efficient long-term tracing of heterogeneous primary cultures. When grafted into the trunk, in contrast to previous studies, hADSC were not found in chondrogenic or osteogenic territories up to E8. Surprisingly, 82.5% of the hADSC were associated with HNK1+ tissues, such as peripheral nerves. Human skin fibroblasts showed a smaller tropism for nerves. In line with other studies, hADSC also adopted perivascular locations. When grafted into the presumptive BA1, 74.6% of the cells were in the outflow tract, the final goal of cardiac neural crest cells, and were also associated with peripheral nerves. This is the first study showing that hADSC could adopt a perineural niche *in vivo* and were able to recognize cues for neural crest cell migration of the host. Therefore, we propose that xenografts of human cells into chick embryos can reveal novel behaviors of heterogeneous cell populations, such as response to migration cues.

## INTRODUCTION

Adult stem cells are essential to maintain tissue homeostasis throughout life. The stromal or “mesenchymal” cells are heterogeneous populations that are a source of progenitors and stem cells (Crisan et al., 2008). The first stromal clonogenic and multipotent cell population was identified by Friedenstein and coworkers in 1966, in the bone marrow, the CFU-F (colony forming units-fibroblastic). These cells gave rise to skeletal derivatives *in vivo* (Friedenstein et al., 1974; Owen and Friedenstein, 1988) and *in vitro* (Pittenger et al., 1999). In the search for an adult cell alternative to embryonic stem cells, the term “mesenchymal stem cell” (MSC) was coined by Caplan, who predicted their potential to give rise to all mesodermal lineages (Caplan, 1991), generating a lasting debate about the true plasticity and role *in vivo* of these cells (Bianco et al., 2008; da Silva Meirelles et al., 2008; Hematti, 2012; Hüttmann et al., 2003; Phinney, 2012; Prockop, 1997).

In addition to bone marrow, some research groups have isolated cells with similar biological properties from different tissues and organs (Crisan et al., 2008; da Silva Meirelles et al., 2006). The distribution of MSC-like cells *in vivo* suggests, in part, an association with blood vessels, such as pericytes or adventitial cells (Corselli et al., 2012; Crisan et al., 2008; Sacchetti et al., 2007). However, identification of the MSC lineage *in vivo* has been delayed by the characterization of these cells by their *in vitro* properties and their ambiguous phenotypic characterization.

Although the International Society for Cell Therapy (ISCT) has proposed guidelines for the characterization and culture of MSC (Dominici et al., 2006; Horwitz et al., 2005), it has been shown that MSC-like cells derived from different tissues are functionally heterogeneous, even at the intra-population level (Bianco et al., 2010; Phinney, 2012; Tallone et al., 2011). Differences in the origin of the MSC lineage during embryonic development, as well as the heterogeneity of the cell population isolated from a specific tissue, may be at the bottom of this issue.

The subcutaneous adipose tissue is also a source of stromal cells (Zuk et al., 2001). The abundance and accessibility of this tissue makes it an attractive source of adult progenitor/stem cells for regenerative medicine (Zuk et al., 2001). Adipose-derived stromal cells (ADSC) contain several subpopulations, including adipocyte precursors (Pettersson et al., 1985; Poznanski et al., 1973), perivascular cells (Bourin et al., 2013; Corselli et al., 2012; Crisan et al., 2008; Tallone et al., 2011; Zimmerlin et al., 2010) and uncharacterized adherent cells (Phinney, 2012).

The subcutaneous adipose tissue of the trunk derives from the mesoderm (Mauger, 1972). However, some evidence indicates that MSC-like subpopulations could be composed by cells derived from the neural crest (Komada et al., 2012; Sowa et al., 2013). For instance, several reports have described glial differentiation of ADSC, both *in vitro* (di Summa et al., 2013; Kaewkhaw et al., 2011; Radtke et al., 2009) and *in vivo*, following grafts in crushed tibial nerves (Tomita et al., 2013) or spinal-cord lesions (Chi et al., 2010). Also, ADSC successfully improved peripheral nerve repair when associated with scaffolds (Widgerow et al., 2014). This raises the question of whether these effects correspond to a still-undescribed role of these cells in tissue homeostasis, and if it might be associated with a specific ADSC subpopulation.

Although previous studies have investigated the capacity for differentiation of ADSC after grafting in adult tissues (James et al., 2012b; Lee et al., 2013; Lendeckel et al., 2004; Mesimäki et al., 2009; Zheng et al., 2006), no study has investigated the behavior of ADSC grafted in a developing embryo. This approach provides a permissive and inductive environment for ADSC, with the potential to reveal their intrinsic migration and differentiation capacities in response to the surrounding cues. More importantly, this approach does not require presumptions about subpopulations or specific differentiation pathways before the cells are grafted. Thus, the embryo can be used as a platform to investigate the plasticity of human cells *in vivo*, in a more inductive environment than the adult organism.

Xenografts of human cells into chick embryos have been performed previously to address different scientific questions. For instance, grafting highly metastatic melanoma cells into the cephalic neural-crest region revealed that the embryonic environment can revert their malignant phenotype (Kulesa et al., 2006). This model would also be useful to study human adult stem cells, as chick embryos allow cells to be grafted in precise territories, are amniotes, and develop at 37°C. To locate the human cells, an ideal technique must be able to identify any donor cell without affecting them prior to the graft, including low-passage primary cultures, in a similar fashion to the quail-chick chimeras (Le Douarin, 1973). *Alu* elements are retrotransposons found exclusively in primates, which comprise 10.6% of the human genome (Cordaux and Batzer, 2009), making them a target for distinguishing human genetic material via *in situ* hybridization, using DNA probes.

After grafting ADSC spheroids into the paraxial mesoderm of E2 (embryonic day 2) chick embryos, we evaluated the migration of human cells under the influence of asymmetric cues that pattern the somites during development (Christ and Scaal, 2009). The region of the presomitic mesoderm medial to the presumptive forelimb was chosen, because this environment is capable of inducing chondrogenesis, myogenesis, endochondral ossification and formation of the dorsal dermis, among other tissues. In addition, events such as trunk neural-crest migration (Le Douarin and Kalcheim, 1999), vasculogenesis (Pardanaud et al., 1996; Pouget et al., 2008) and intraembryonic haematopoiesis (de Bruijn et al., 2000) take place in this region, allowing us to understand how the hADSC will behave regarding them.

Here, we demonstrate that *in situ* hybridization with *Alu* probes is an efficient method to localize human cells in chick embryos at middle stages of development such as E8, without requiring prior manipulation such as gene transfection or the use of tracer dyes. In addition, we observed for the first time that the adult human ADSC, when grafted in the embryonic environment, could recognize the host neural-crest cell migration pathways, and were found preferentially associated with the peripheral nervous system. Thus, this model provides a new perspective on ADSC, highlighting the importance of further investigations of this heterogeneous population that has many potential uses in cell therapy.

## RESULTS

### Genomic *in situ* hybridization using DNA probes for human *Alu* sequences did not cross-react with chick DNA

Digoxigenin-labeled DNA probes were synthesized via PCR using DNA from human adipose-derived stromal cells (hADSC) as a template (Steck et al., 2010). The length of the main amplification product was 250–300 bp (supplementary material Fig. S1), as predicted. To test the specificity of the probes, *in situ* hybridization with *Alu* probes was performed in different human cell types: hADSC, bone marrow-derived stromal cells, MCF-7 (supplementary material Fig. S1), human-skin fibroblasts, U87 and A549 (data not shown). The nuclei of all human cells were positively stained. Cross-sections of an E6 chick embryo were used as a negative control (supplementary material Fig. S1), and no stained nuclei were found. These data confirm that genomic *in situ* hybridization with *Alu* probes is a specific method to identify human cells, and efficiently stains both cell lineages and low-passage, heterogeneous primary cell populations.

### hADSC grafted into E2 (HH11-12) chick embryos could be localized using *Alu* probes

**Fig. 1.**
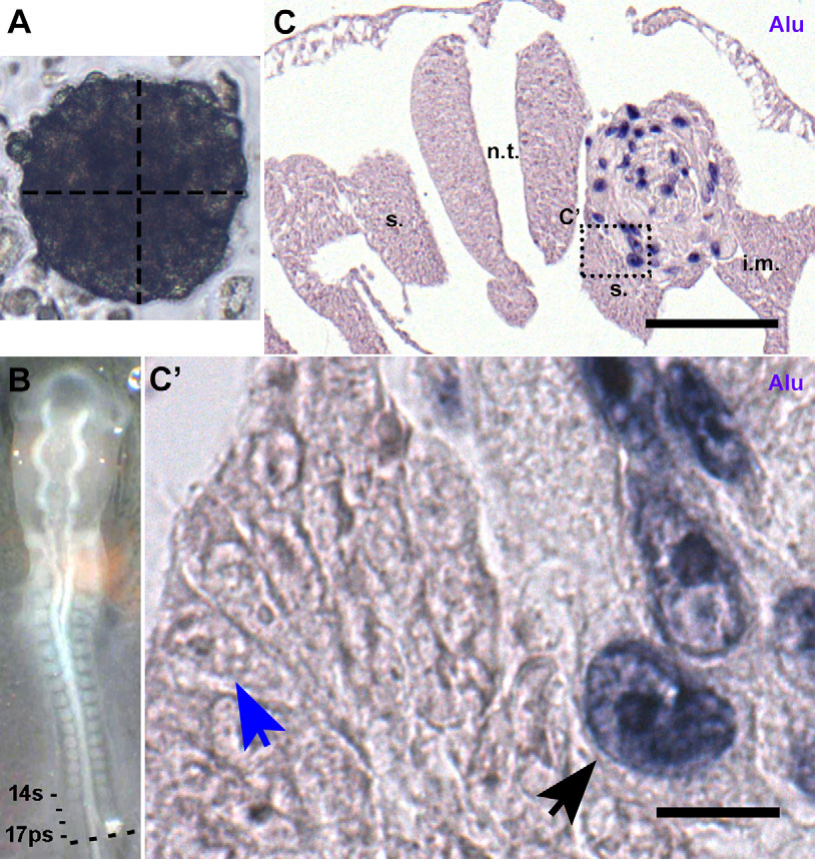
**Grafting hADSC in 2-day chick embryos.** (A) Spheroid suitable for grafting. Vertical line: 131.4 µm; horizontal line: 130.8 µm. (B) 14ss embryo with a spheroid grafted at presumptive 17th somite level. (C) Cross-section of the embryo in B (dashed line), 4 h after surgery. *In situ* hybridization with human *Alu* probes. The hADSC spheroid did not affect the formation of chick tissues. Scale bar: 100 µm. (C′) Only human nuclei (black arrow) were stained after *in situ* hybridization; chick nuclei (blue arrow) and human cytoplasm were unstained. Scale bar: 10 µm. i.m., intermediate mesoderm; n.t., neural tube; s., somite.

In order for xenografting of human cells into chick embryos to serve as an adequate model, it must not disrupt the host's morphogenesis and it must allow localizing a single donor cell. Spheroids were grafted instead of injecting a cell suspension into the embryo, which ensured precise placement of the graft and allowed us to investigate cell migration. hADSC spheroids were prepared 2 days before the surgery (Brito et al., 2008), yielding spheroids of numerous sizes. Spheroids with the approximate size of a somite were chosen by visual inspection. They contained a mean of 52.3 cells (*n*=10) and had a mean diameter of 135 μm (*n*=5) (Fig. 1A). Embryos developed until somite stage (ss) 13–19 [Hamburger and Hamilton (HH) stage 11–12; Hamburger and Hamilton, 1951], when a spheroid was grafted laterally to the neural tube in the presomitic mesoderm at the wing-bud level (presumptive somites 15–21) (Fig. 1B).

Four hours later, the integration of the human cells within the host tissue was analyzed in cross-sections (dashed line, Fig. 1B) (*n*=2). *In situ* hybridization with *Alu* probes was highly specific for human cells in the spheroid, and did not stain the chick tissues (Fig. 1C). The hADSC were still aggregated and were located in the mesoderm between the neural tube and the intermediate mesoderm (Fig. 1C). At higher magnification (Fig. 1C′), only the nuclei of hADSC cells showed dark-blue staining, indicative of *Alu* sequences (Fig. 1C′, black arrow), while the nuclei of chick cells were unstained (Fig. 1C′, blue arrow). Neither human nor chick cells showed cytoplasmic staining. Typical embryonic tissues were found at the correct location on both the control and the grafted sides. Thus, human hADSC could be localized with *Alu* probes in the embryo with the same efficiency observed in cell cultures.

### hADSC integrated into the chick embryo and migrated at E3.5 (HH21-22)

The first question addressed was whether the human cells would remain aggregated, or would integrate with local chick tissues and migrate alongside other cell populations of the embryo. Chick embryos grafted with hADSC were fixed at E3.5, and the cell location was determined in cross-sections at the wing-bud level.

**Fig. 2.**
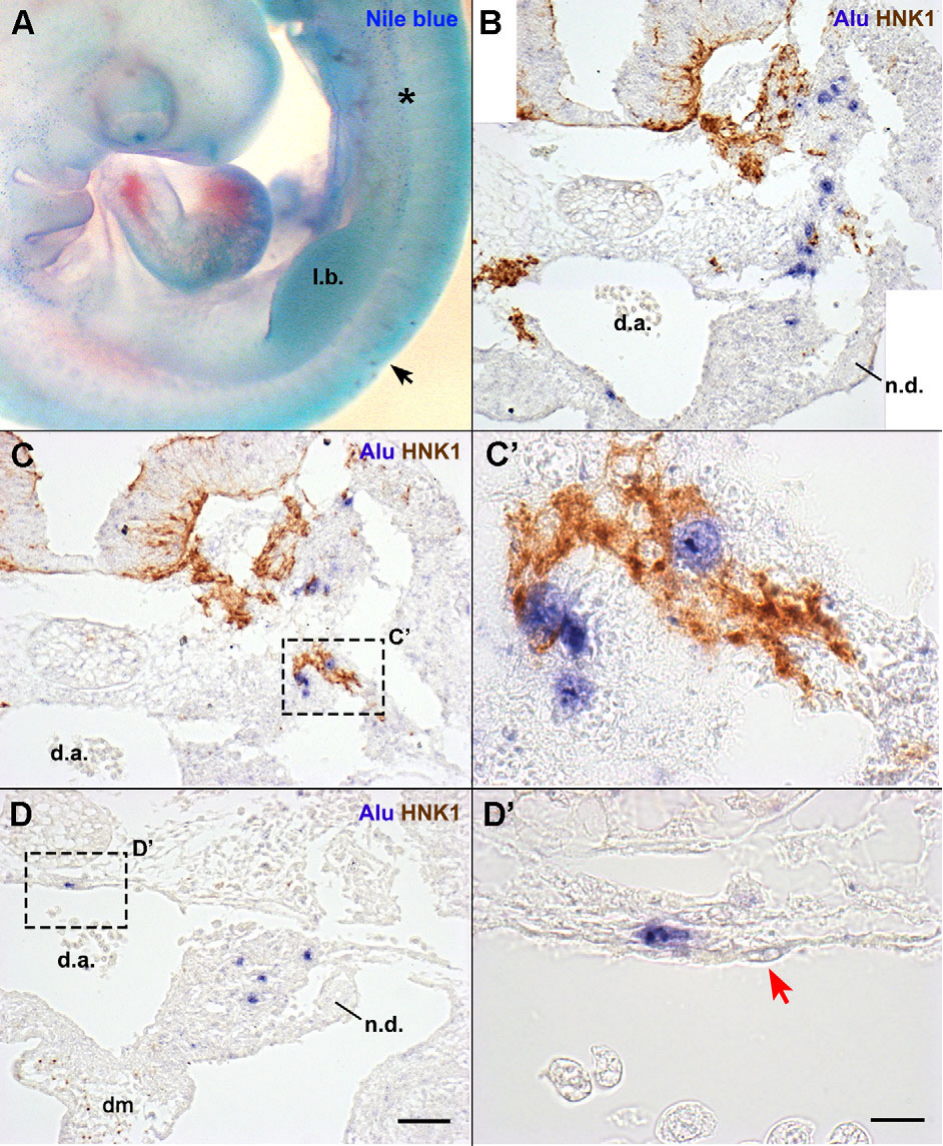
**Localization of hADSC in E3.5 chick embryos.** (A) Nile blue sulfate staining revealed that, in the operated region (black arrow), there was no increase in cell death or apparent malformations in the embryo. Cell death is represented by the blue puncta, as observed in the mesonephros (asterisk). (B,C,D) Cross-sections at the anterior limb bud level of the embryo in A. Sections were hybridized with *Alu* probes and immunostained with HNK1 (migratory NCC). The spheroid was no longer visible, and human cells integrated with the embryo. (B) Human nuclei were observed in the mesenchyme from the neural tube until the mesonephros. (C,C′) Some human cells mingled with the NCC migration stream. (D) Cells observed in the mesonephros and dorsal aorta (d.a.) did not associate with NCC at this stage. (D′) hADSC associated with the d.a. were always in a perivascular position. The endothelium was derived from chicken (red arrow). B,C,D, scale bar: 50 µm; C′,D′, scale bar: 10 µm. l.b., wing limb bud; d.m., dorsal mesentery; n.d., nephric duct.

First, cell death was investigated using Nile blue sulfate, a vital stain (Jeffs and Osmond, 1992). No cell death beyond the levels reported for normal development (Hirata and Hall, 2000; Jeffs and Osmond, 1992) was observed at E3.5 (*n*=3) (Fig. 2A). Few cells were stained with Nile blue sulfate in the vicinity of the graft (Fig. 2A, black arrow), suggesting that the surgery and the grafted spheroids did not induce cell death in the tissues of the chick embryo.

Histological sections revealed a similar development on both the experimental and the control sides, with no observable malformations induced by the surgery. In E3.5, *Alu*+ cells were found either in the mesenchyme or in the perivascular region (Fig. 2). Most of the cells (83.6%, *n*=2/2) were located in the mesenchyme lateral to the neural tube, distributed along its entire dorsoventral extension (Fig. 2B,C). Other cells, however, were found in the aorta-gonad-mesonephros region (Fig. 2B,D), in the mesonephros mesenchyme (6.6%, *n*=2/2), the dorsal mesentery (2.0%, *n*=1/2), or perivascular to the dorsal aorta (7.9%, *n*=2/2). The perivascular *Alu*+ cells (Fig. 2D′) were observed in contact with the chicken dorsal-aorta endothelium (Fig. 2D′, red arrow). *Alu*+ cells were not found in the limb buds, ventral to the dorsal mesentery, or forming the nephric-duct epithelium or endothelium.

Since hADSC were grafted in the somite region, we investigated if the migration of neural-crest cells (NCC) was disturbed, as well as their interaction with human cells. Migratory NCC, identified by the HNK-1 antibody (Tucker et al., 1984), were observed equally on both the operated and non-operated sides of the embryo (data not shown). Interestingly, part of the hADSC located in the mesenchyme lateral to the neural tube and notochord appeared closely associated with NCC of the ventral migratory pathway (Fig. 2C′). Collectively, these data indicate that hADSC not only did not induce malformations in the chick embryo, but survived in the chick embryonic microenvironment and became integrated with the host tissues.

### hADSC were not located in the presumptive chondrogenic, osteogenic or myogenic territories of E6 (HH29) chick embryos

**Fig. 3.**
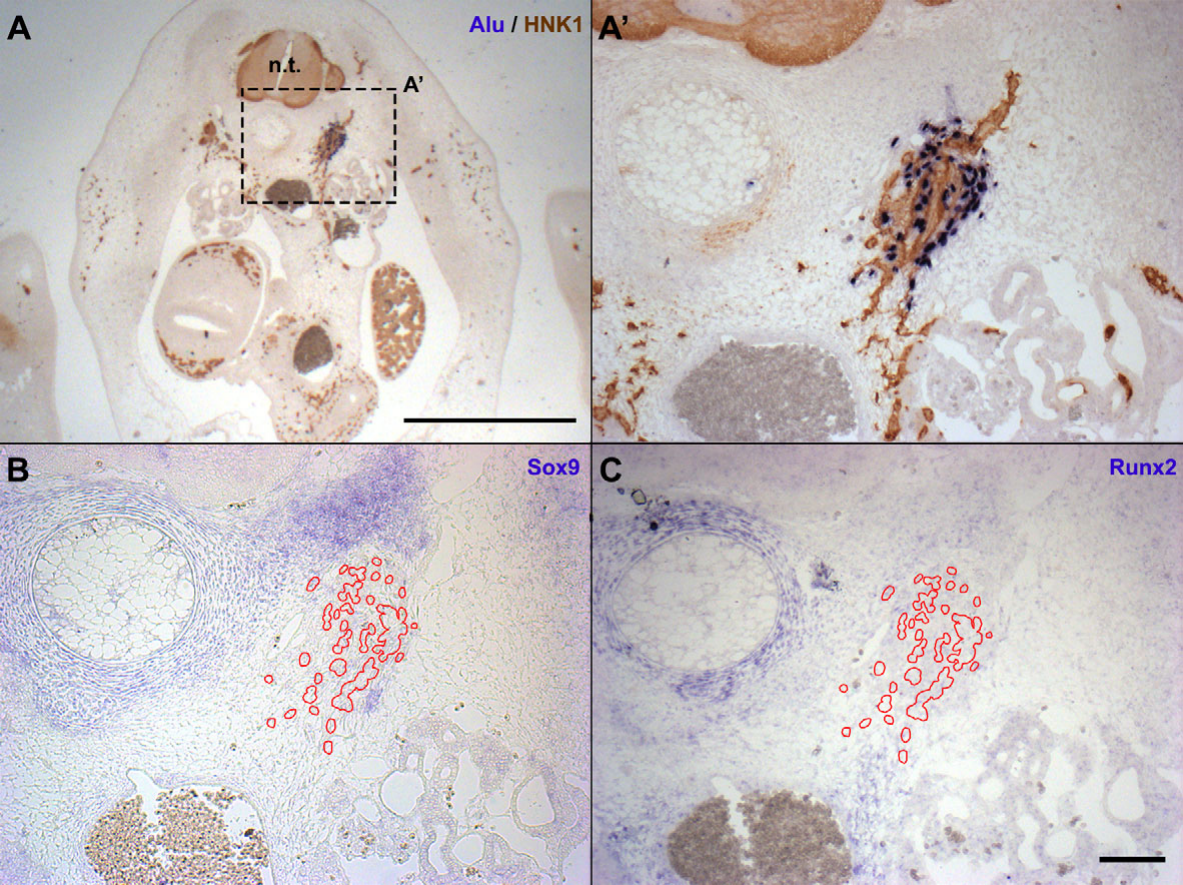
**hADSC were not localized in osteogenic or chondrogenic regions in E6 chick embryos.** (A,A′) Cross-sections at the level of the anterior limb bud hybridized with *Alu* probes revealed a group of human cells lateral to the vertebral condensation. The nerve was revealed with HNK1 immunostaining. (B,C) *In situ* hybridization with chicken RNA probes in sections serial to (A′). hADSC (red outlines) were not found in chondrogenic (B) (*Sox9*+) or osteogenic (C) (*Runx2*+) regions. A, scale bar: 1 mm; A′,B,C, scale bar: 100 µm. n.t., neural tube.

At E6, the advanced stage of organogenesis allowed us to investigate the behavior of hADSC regarding committed territories and differentiating tissues. It has been reported that hADSC can differentiate into skeletal derivatives *in vitro* (Zuk et al., 2001) and *in vivo* (James et al., 2012b; Lendeckel et al., 2004; Mesimäki et al., 2009; Zheng et al., 2006). Therefore, we analyzed whether hADSC would be located in chick territories committed to chondrogenesis and osteogenesis. We also investigated whether hADSC would be found in myogenic territories, another mesoderm-derived tissue. *In situ* hybridization with *Alu* probes was performed in parallel with Alcian blue staining, to reveal cartilage; and *in situ* hybridization with *Sox9, Runx2* and *MyoD* probes for chick RNA was performed to identify, respectively, cartilage-, bone- and muscle-committed territories of the host (*n*=3) (Fig. 3).

The *Sox9*-positive, Alcian blue-positive chondrogenic territories around the neural tube were always devoid of *Alu*+ cells (Fig. 3A,B). Although ossification had not yet started in the trunk at E6, the presumptive osteogenic region could be detected by *in situ* hybridization for *Runx2* (*Cbfa1*). At E6, no *Alu*+ cells were found in the mesenchyme expressing *Runx2* (Fig. 3C), which is associated with territories undergoing endochondral ossification. The *Alu*+ cells were also absent from the mesenchyme expressing *MyoD* in the trunk (*n*=3), except for a few cells associated with the wing-bud nerve, in a *MyoD*+ domain (data not shown). Therefore, in contrast to the potential reported in the literature, hADSC were not associated with chondrogenic, osteogenic or myogenic regions until the sixth day of development.

### hADSC were associated with peripheral nerves and blood vessels of E6 (HH29) and E8 (HH34) chick embryos

**Fig. 4.**
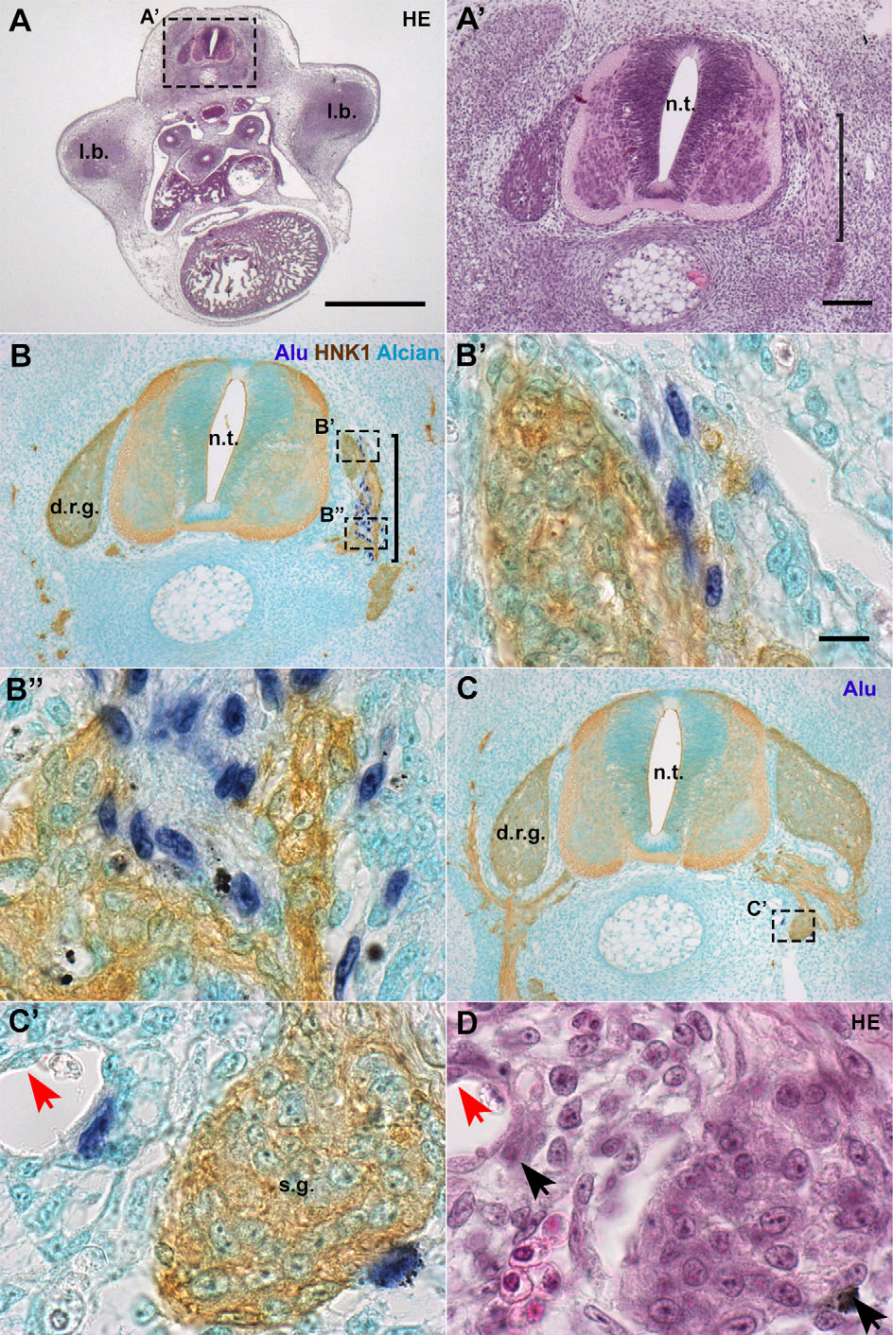
**In E6 chick embryos, hADSC associated with peripheral nerves and ganglia.** (A,A′) Cross-sections at anterior limb-bud (l.b.) level revealed that the morphology of the embryo is normal. Haematoxylin-eosin (HE) staining. (B,B′,B″) In serial sections, hybridization with *Alu* probes performed simultaneously with HNK1 immunostaining revealed that hADSC were closely associated with developing nerve fibers, lateral to the neural tube (n.t.), in the region of the dorsal-root ganglion (d.r.g.). Alcian blue counterstaining. (C,C′) hADSC were associated with the sympathetic ganglia (s.g.). (D) Cells were also associated with blood vessels, always in a perivascular location, as seen with HE staining. Red arrows: endothelium. A, scale bar: 1 mm; A′,B,C, scale bar: 100 µm; B′,B″,C′, scale bar: 10 µm.

Given the unexpected results described above, it was necessary to characterize the distribution of hADSC and whether they interacted with specific cell types of the chick embryo. First, cross-sections hybridized with *Alu* probes were compared with adjacent sections stained with haematoxylin-eosin (HE). E6 embryos had normal morphology (Fig. 4A). In *Alu*-hybridized sections, positive cells were observed lateral to the neural tube in the region of the dorsal-root ganglia and sympathetic chain (Fig. 4A′,B, brackets).

To investigate the morphology of the nerves and their relationship to the hADSC, we used the HNK1 antibody, which recognizes both glial and neuronal cells of the peripheral nervous system (PNS) in this stage (Vincent and Thiery, 1984). In humans, HNK1 is present in peripheral nerves, in a pattern similar to the anti-MAG (Myelin-Associated Glycoprotein) antibody (Inuzuka et al., 1984), including in human fetal Schwann cell culture (Kim et al., 1989). *Alu*+ nuclei were closely associated with HNK1+ cells (Fig. 4B,C), revealing that human cells were associated with both the dorsal-root ganglia (DRG) (Fig. 4B′) and their projections (Fig. 4B″) (*n*=3/6, see Table 1). *Alu*+ cells were also found associated with the sympathetic chain (Fig. 4C,C′) (*n*=5/6). Moreover, few *Alu*+ cells had migrated dorsally and were located close to the roof plate of the neural tube (*n*=2/6) (data not shown), but never integrated into the neuroepithelium. In addition to their association with nerves, an interaction of hADSC with blood vessels was also noted (Fig. 4C′). *Alu*+ cells appeared in close contact with endothelial cells in a perivascular position, but never composing the endothelium (Fig. 4D, red arrow).

**Table 1.**
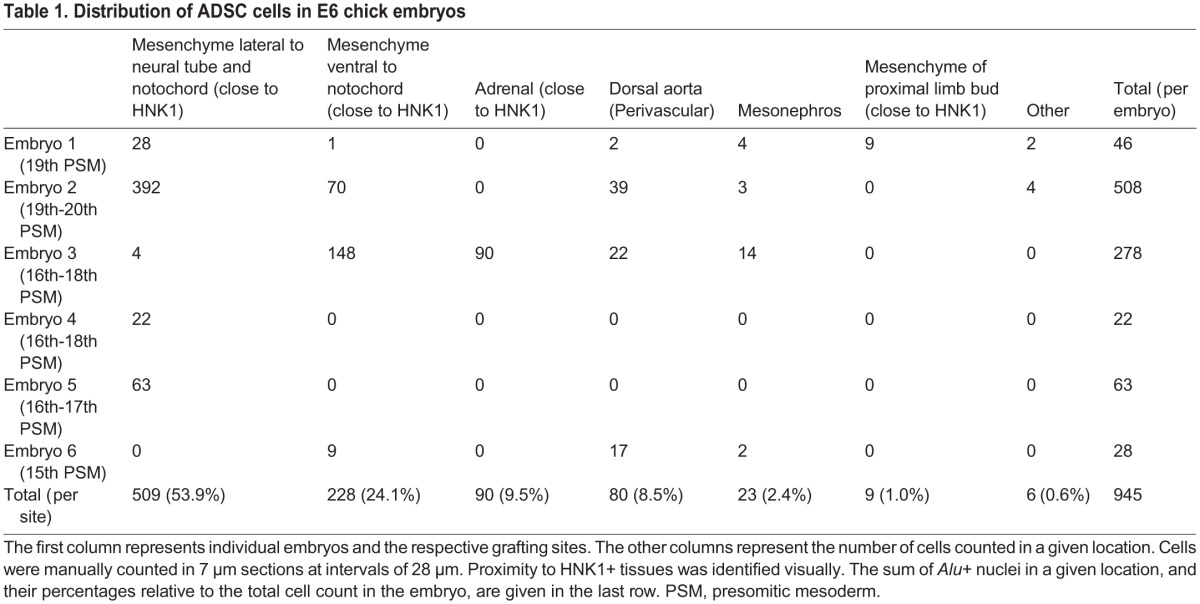
**Distribution of ADSC cells in E6 chick embryos**

**Fig. 5.**
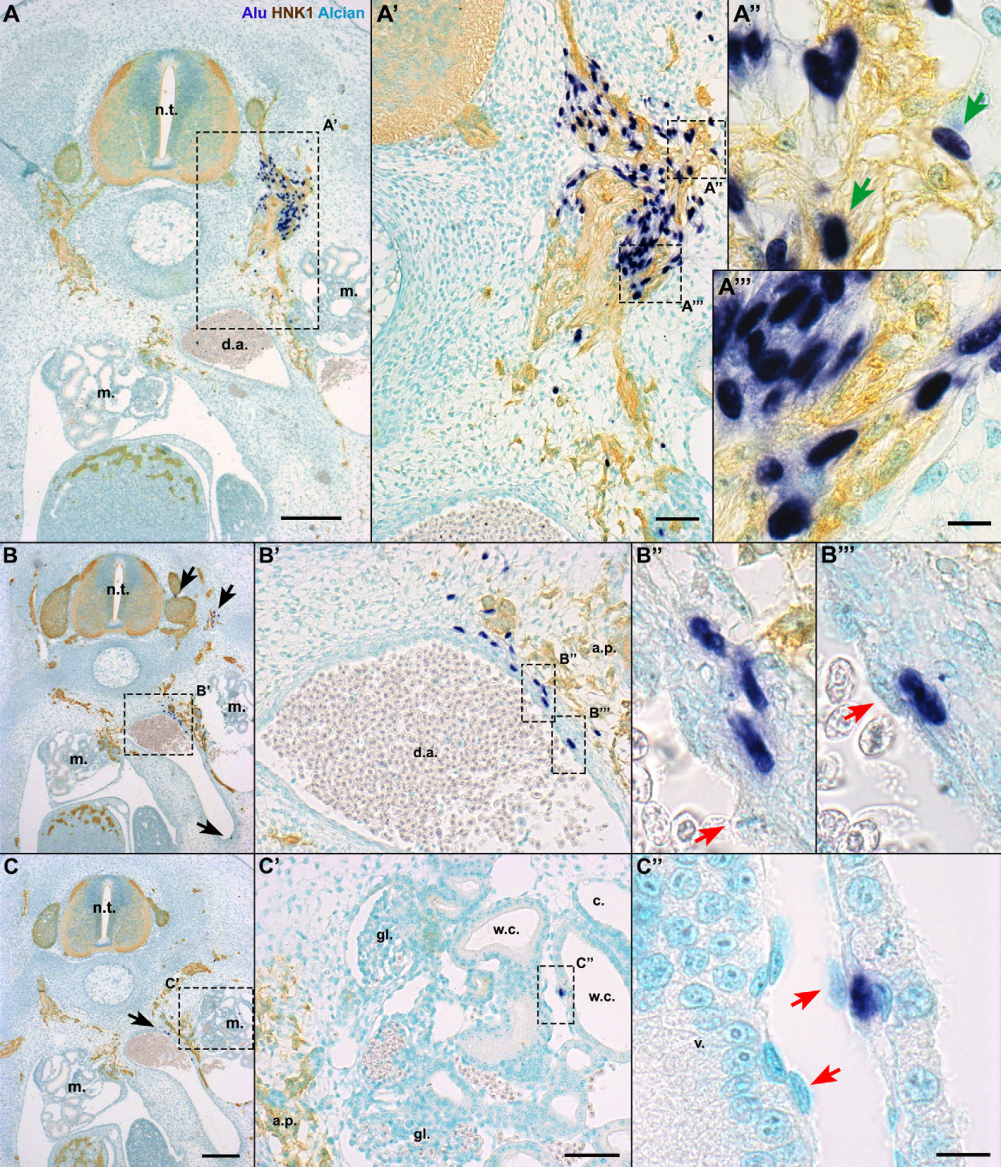
**In E6 chick embryos, hADSC migrated associating with peripheral nerves and also formed blood vessels.** *In situ* hybridization with *Alu* probes, immunostaining with HNK1 antibody and Alcian blue counterstaining in cross-sections at the anterior limb bud level. (A,A′) When hADSC were grafted on the level of the aortic plexus (a.p.), human cells were found ventrally until the dorsal aorta (d.a.), associated with HNK1+. A few cells presented a HNK1+ cytoplasm (A″, green arrows), although most of them were HNK1− (A‴). (B) hADSC were located in the d.a. wall. Note other cells lateral to the neural tube (n.t.) and in the ventralmost region of the d.a. (black arrows). (B′,B″,B‴) Human cells were perivascular, while the endothelium was chicken-derived (red arrows). (C,C′) hADSC were found in the mesonephros (m.). (C″) Human cells were in contact with the basal lamina of the Wolffian channels (w.c.), in a perivascular location. Again, the endothelium was derived from chicken. A,B,C, scale bar: 200 µm; A′,B′,C′, scale bar: 50 µm; A″,A‴,B″,B‴,C″, scale bar: 10 µm. c., coelom; gl., glomerulus; v., blood vessel.

In levels where the nerves extended farther than the neural tube and the sympathetic chain, the hADSC had the capacity to migrate along these nerves into additional regions of the embryo (*n*=4/6). The distribution of *Alu*+ cells from the dorsal region of the neural tube to the mesonephros and ventral side of the dorsal-aortic wall is shown in a series of cross-sections (Fig. 5A,B,C, black arrows). The more-ventral distribution of human cells was closely associated with the aortic plexus, as shown by the association of *Alu+* cells with HNK1+ nerves extending to the dorsal aortic walls (Fig. 5A′,B′). Reinforcing the notion that hADSC distribution could be related to peripheral nerves and blood vessels, in cross-sections where the aortic plexus was not observed, *Alu*+ cells remained more dorsal, at the level of the sympathetic chain (Fig. 4C,C′) (*n*=2/6).

Some *Alu*+ cells integrated into the wall of the dorsal aorta (8.5%, *n*=4/6), either directly associated with the endothelium (Fig. 5B‴) or in an adventitial position (Fig. 5B″). Having in mind that hADSC were reported to adopt both niches in the adult tissue (Zimmerlin et al., 2010), we performed immunostaining with alpha-smooth muscle actin (SMA) to determine if the hADSC adopted a pericytic (SMA+) or adventitial (SMA−) position. Of the total number of human cells of embryos evaluated for SMA (*n*=3), only 2.3% (7 of 299 cells) colocalized with SMA (supplementary material Fig. S3A′,B). The others were perivascular to smaller vessels (SMA−) or in contact with SMA+ cells, but in an adventitial position (18.3%, 55 of 299 cells) (supplementary material Fig. S3C′,D).

Some human hADSC were observed in the mesonephros close to the Wolffian ducts (2.4%, *n*=4/6) (Fig. 5C′). A higher magnification revealed that these cells were also in a perivascular location between the endothelial cells (red arrows) and the epithelia of the Wolffian ducts (Fig. 5C″). The *Alu*+ nuclei never formed the mesonephric epithelium itself.

In one embryo, human hADSC migrated to the region between the dorsal aorta and mesonephros, where they located in the developing adrenal gland (supplementary material Fig. S2). The *Alu*+ nuclei were observed in the neighborhood of the *Bmp4*-expressing adrenal primordium (Huber et al., 2008), in association with HNK1+ sympathoadrenal cells (supplementary material Fig. S2). In another embryo, where a nerve branch projected into the limb bud at the level of the graft, a few hADSC were observed in the proximal limb-bud region associated with an HNK1+ nerve (data not shown) (Table 1).

The surprising association between hADSC and HNK1+ tissues raised the question of whether the human cells were differentiating into Schwann cells and sympathoadrenal progenitors, or were only interacting with these tissues. Therefore, the number of *Alu*+ nuclei that colocalized with HNK1+ cytoplasm (Fig. 5A″) was quantified, in contrast to the cells that were clearly perineural and did not express HNK1+ (Fig. 5A‴). In E6 embryos (*n*=6), 5.9% (56 of 945 cells) of all human cells appeared to colocalize with HNK1+ staining, while 82.5% (780 of 945 cells) were perineural, adjacent to HNK1+ cells. This suggests that a minor part of the hADSC adopted fates typical of neural-crest derivatives, but the majority was sharing a perineural niche.

In E8 embryos (*n*=3), hADSC were located in regions previously identified in E6 embryos. Cells were observed ventral to the sympathetic ganglion, associated with a HNK1+ ganglion-like structure close to the dorsal aorta, or associated with small blood vessels (*n*=2/3) (supplementary material Fig. S3). Cells were also found between the vertebral condensation and the neural tube, in the area of the meninx (*n*=1/3) (supplementary material Fig. S3). Human cells were localized in the region of the dorsal root, enriched adjacent to HNK1+ fibers. Thus, in both E6 and E8, hADSC did not locate in skeletal or muscle territories, but did locate in close association with nerves and blood vessels.

### Human-skin fibroblasts grafted in the same region as hADSC revealed a smaller tropism for nerves in E6 (HH29) chick embryos

It was necessary to determine the degree to which the behavior of the hADSC was dependent on their intrinsic properties, versus the degree to which it was controlled by microenvironment signaling factors. It has been previously reported that human embryonic stem cells grafted in the same region do not adopt perineural nor perivascular niches. Therefore, we decided to graft another primary stromal-cell population, human-skin fibroblasts (hSF) derived from the foreskin, into the same region.

**Fig. 6.**
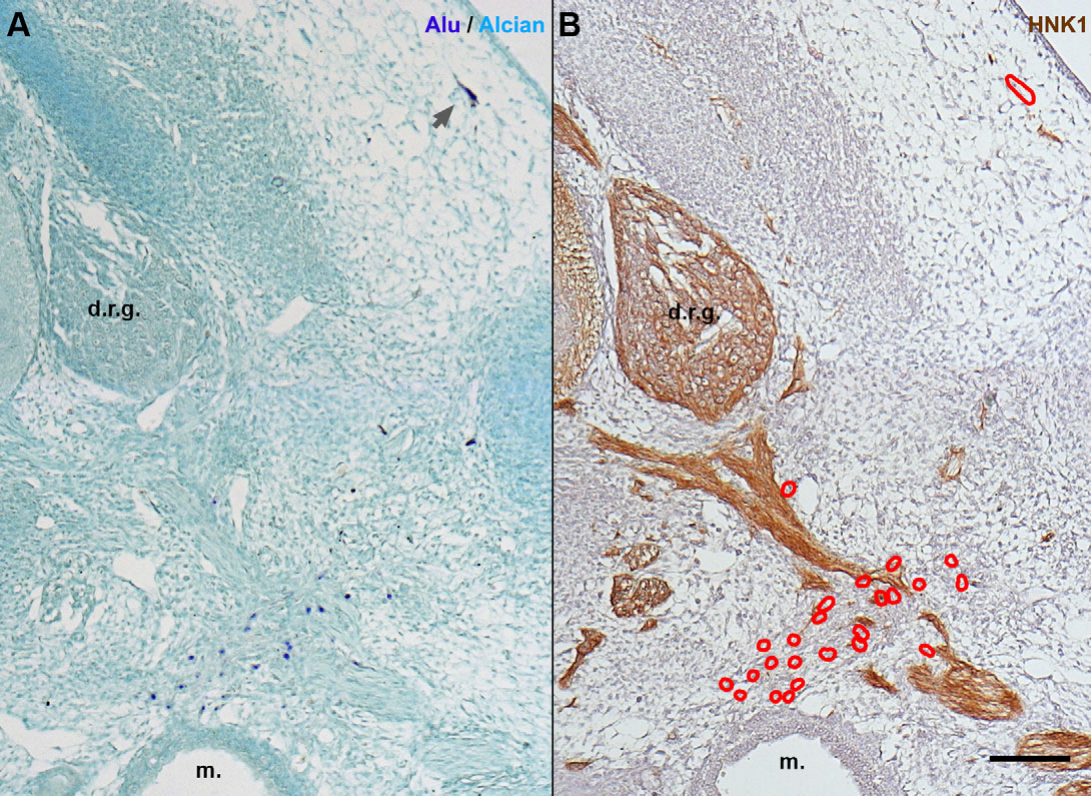
**In E6 chick embryos, hSF revealed a smaller tropism for nerves than hADSC.** (A) *In situ* hybridization with *Alu* probes and Alcian blue counterstaining in cross-sections at anterior limb bud level. hSF were dispersed throughout the mesenchyme (dark blue nuclei), including the dermis (gray arrow). (B) In an adjacent section immunostained with HNK1, the majority of hSF (red outlines) were not adjacent to peripheral nerves. A,B, scale bar: 100 µm. d.r.g., dorsal root ganglion; m., mesonephron.

Spheroids of hSF were grafted into the presomitic mesoderm of E2 (15–19ss) chick embryos, and the cells were located using *Alu* probes. At E6, the hSFs were dispersed throughout the embryo (*n*=4), showing different distributions (Fig. 6). In contrast to hADSC, 5.1% of the hSFs were able to migrate into the mesenchyme of the dermis (*n*=4/4) (Fig. 6A, gray arrow), adopting a more elongated, fibroblast-like phenotype. Moreover, part of the hSF were observed dispersed throughout the mesenchyme, without either perivascular or perineural locations (44.2%, *n*=4/4). Similar percentages of hSF (16.2%, *n*=4/4) and hADSC (10.9%) were found in perivascular regions. However, only 43.8% (*n*=4/4, 80 of 235 cells) of the hSF were observed associated with HNK1+ tissues, in contrast to the 88.5% (*n*=6/6, 836 of 945 cells) of the hADSC that occupied this niche. No hSF appeared to be HNK1+. The distribution of hSF in E6 is described in supplementary material Table S1.

### hADSC grafted into the presumptive first branchial-arch region associated with the peripheral nervous system and host cardiac neural-crest cells

Since hADSC were not able to integrate into the mesenchyme derived from mesoderm, but instead interacted with host tissues derived from neural-crest cells (NCC), we addressed whether the hADSC would integrate into the NCC-derived ectomesenchyme of the first branchial arch (BA1). Therefore, hADSC spheroids were grafted into the presumptive BA1, between the ectoderm and endoderm lateral to the boundary of the mesencephalon and the first rhombomere of 5–8ss chick embryos, as previously described (Brito et al., 2008). hADSC were then identified in coronal sections of E4.5 chick embryos (*n*=3).

**Fig. 7.**
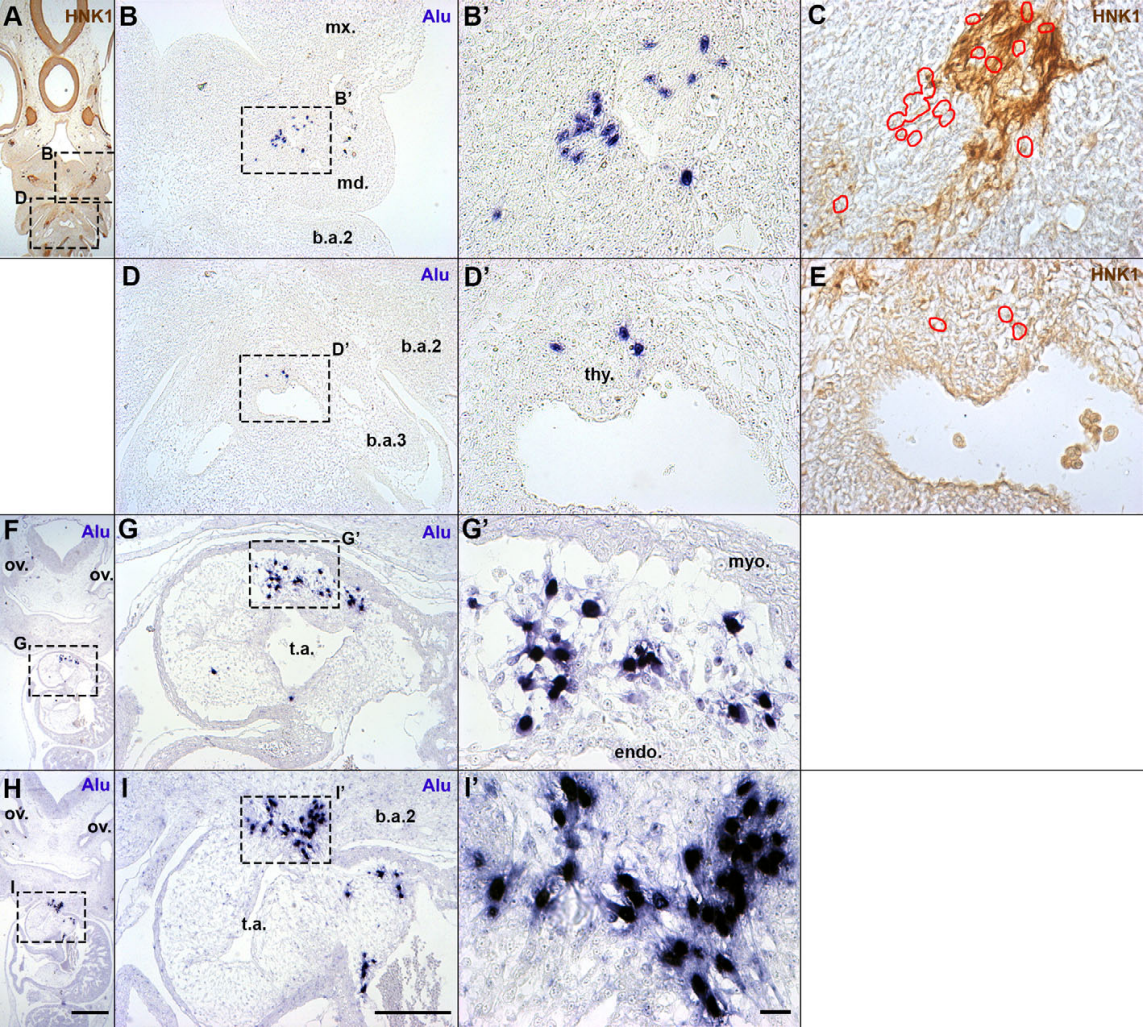
**When grafted in the first branchial arch (BA1) presumptive region, hADSC migrated as neural crest cells.** (A,F,H) Coronal sections of E4.5 chick embryos. Scale bar: 500 µm. ov., otic vesicle. (B,B′,C) The hADSC found in BA1 derivatives were in the mandibular bud (md.), associated with HNK1+ nerves. Red outlines: location of *Alu*+ nuclei in serial sections. (D,D′,E) Few hADSC were observed around the thyroid bud (thy.). (G,G′) Most of the hADSC migrated alongside cardiac neural crest cells and were found in the truncus arteriosus (t.a.). hADSC were in the cardiac jelly, between the endocardium (endo.) and myocardium (myo.). (I,I′) It was possible to observe hADSC dispersed from the second branchial arch (b.a.2) into the t.a. B,D,G,I, scale bar: 200 µm; B′,C,D′,E,G′,I′, Scale bar: 20 µm. b.a.3, third branchial arch; mx., maxillary bud; ph., pharynx.

Part of the hADSC was found in a BA1 derivative, the mandibular bud (21.4%) (*n*=2/3) (Fig. 7A,B). *Alu*+ cells were not dispersed through the mesenchyme; HNK1 immunostaining in adjacent sections revealed that human cells were associated with cranial nerves (Fig. 7B′,C), in a behavior similar to the hADSC grafted into the trunk.

Surprisingly, most of the cells were found composing the mesenchyme of the outflow tract, in the truncus arteriosus region (74.7%) (*n*=3/3) (Fig. 6F,H). The cells were in the mesenchyme between the myocardium and endocardium (Fig. 7G,G′). It was possible to observe cells dispersing from the second branchial arch ventral region (2.2%) into the aortic arch and truncus arteriosus (Fig. 7I,I′). The pattern of hADSC distribution in the heart was similar to the cardiac NCC distribution in this stage (Waldo et al., 1998). Additionally, a few human cells were observed around the thyroid primordium (1.1%) (*n*=1/3) (Fig. 7D,D′,E).

Therefore, hADSC were able to integrate with the craniofacial region of the developing embryo. The pattern of hADSC migration reinforced the idea that part of these cells can recognize signals important for the cardiac NCC and peripheral-nerve development of the host.

## DISCUSSION

In this study, we developed a model that allows study of the behavior of human adipose-derived stromal cells (hADSC) in the chick embryonic environment. Using DNA probes for *Alu* sequences, it was possible to follow the distribution of hADSC after engraftment into E2 chick embryos. After being grafted, most of the hADSC associated with the migratory neural crest cells (NCC) and closely interacted with developing nerves. Notably, other hADSC became perivascular to blood vessels of various calibers. Moreover, hADSC were not found in mesoderm-derived tissues such as chondrogenic (*Sox9+*), osteogenic (*Runx2+*) and myogenic (*MyoD+*) territories or in the dermal region. Human-skin fibroblasts (hSF), on the other hand, were able to migrate to the dermis and showed fewer cells associated with nerves. Although the hSF also adopted a perivascular niche, a considerable part of the cells were in the mesenchyme, not perineural or perivascular. When Goldstein et al. (2002) grafted human embryonic stem cells (hESC) in the same region, the hESC formed epithelial structures and adopted neither a perineural nor a perivascular location. Taken together, these observations reinforce the idea that the capacity of each cell population to read the same signals emanating from the embryonic microenvironment is specific and is related to their intrinsic properties.

### *In situ* hybridization for human *Alu* sequences is an efficient method to identify human cells grafted into the chick embryo

*In situ* hybridization with *Alu* probes was first used to identify human xenografts by Jacobsen and Daly (1994), who successfully located human glioblastoma cells in mice. Later, several groups used the same strategy to identify human cells (Brüstle et al., 1998; Hatano et al., 1998; Just et al., 2003; Kasten et al., 2005; Steck et al., 2010; Warncke et al., 2004). Although it is extensively used in rodents, this method is applied here to chick embryos for the first time.

The advantages of chick embryos as a host in xenograft studies have been explored in recent decades, as chick embryos can be manipulated at very early developmental stages, and the fate of grafted cells can be evaluated over the most appropriate time period for a given scientific question (reviewed by Davey and Tickle, 2007). Grafts of human cells into chick embryos have been performed previously, using ESC (Goldstein, 2010), tumor cells (Cage et al., 2012; Carter et al., 2012; Cretu et al., 2005; Kasemeier-Kulesa et al., 2008; Kulesa et al., 2006) or, more recently, induced pluripotent stem cells (iPSC) (Amoroso et al., 2013) and even human fetal tissues (Chao et al., 2013). In the majority of these studies, human cells were located using fluorescent reporters. This strategy allows real-time tracing and live imaging; however, it may have disadvantages regarding transfection efficiency and stability, which are very important when using primary cultures and performing long-term tracing experiments. Considering that hADSC are heterogeneous, it is essential to identify the largest number of cells in order to interpret their individual behavior; thus, we chose a technique that located the human cells only after the tissue had been fixed, and consequently did not affect cell behavior and viability, or lose intensity over time.

*In situ* hybridization with *Alu* probes was capable of identifying individual hADSC even 6 days after the graft, in E8 chick embryos. The clear nuclear staining also indicates that the human cells were alive and integrated into the host tissue, since cytoplasmic staining could indicate human DNA phagocyted by host macrophages (Warncke et al., 2004). Therefore, this technique, combined with the amenability of the chick embryo to manipulation, makes human xenografts in the chick an excellent model to study heterogeneous populations of adult human stem cells *in vivo*.

### Fate of hADSC grafted into the chick embryo at the trunk level

In order to begin deciphering hADSC properties, the behavior of the human cells was compared with that of the host structures. Grafting a spheroid with the size of a somite exposed the hADSC to the different signals emerging from adjacent embryonic structures, which pattern the cell fate of somites, and to other cells such as the migratory NCC.

First, the hADSC migration pattern was evaluated in comparison with somitic derivatives. No hADSC were found in the sclerotome-derived chondrogenic (*Sox9+*, Alcian blue+) or osteogenic (*Runx2+*) mesenchyme of E6 embryos (Fig. 3). This contrasts with the widely accepted chondrogenic and osteogenic potential of ADSC observed both *in vitro* (Bourin et al., 2013; Zuk et al., 2001) and *in vivo* (Lendeckel et al., 2004; James et al., 2012b; Mesimäki et al., 2009; Zheng et al., 2006). Furthermore, it has been reported that the osteogenic potential of ADSC is enhanced by treatment *in vitro* with *Sonic Hedgehog* (James et al., 2012a) or *Noggin* (Fan et al., 2013), notochord-derived factors. However, it is important to consider that the microenvironment of the adult bone is distinct from the embryonic sclerotome, and that without the proper scaffold the ADSC does not form bone, even in the adult (Lee et al., 2013).

No hADSC were found in the dermomyotome of E3.5 embryos or forming the dermis, skeletal muscle or endothelium of E6 and E8 embryos. Their absence from the dermis is notable, since hADSC were isolated from abdominal subcutaneous adipose tissue, derived from the somatopleure (Mauger, 1972). However, hSF isolated from foreskins, which are also derived from the somatopleure, were able to migrate into the dermal region, suggesting differences in cell composition between hADSC and hSF. This reinforced the hypothesis that stromal or “mesenchymal” cell populations are not equivalent (Bianco et al., 2008).

It has been suggested that cells with an hADSC phenotype *in vivo* occupy a perivascular niche (Corselli et al., 2012; Crisan et al., 2008; James et al., 2012b) and compose between 30% and 60% of the viable cells found in the stromal vascular fraction of the lipoaspirate (Bourin et al., 2013). When grafted into the embryo, a part of the hADSC was recruited by the developing blood vessels. Interestingly, they were distributed in regions where the perivascular cells originate from the sclerotome, including the dorsal portion of the trunk, blood vessels of the mesonephros, and wall of the dorsal aorta (Jaffredo et al., 2010; Pouget et al., 2008; Wiegreffe et al., 2009).

In the adipose tissue, perivascular progenitor cells that express mesenchymal markers (CD90+, CD34+, CD31−) can be found in contact with the endothelium, where they express α-smooth muscle actin (SMA), or in the outer adventitial ring, comprised of SMA− cells (Zimmerlin et al., 2010). Here, only 2.3% of the hADSC was SMA+, while perivascular SMA− cells represented 18.3% of the hADSC found in the embryo (supplementary material Fig. S3). These results concord with the description for the adult, *in vivo*, by Corselli et al. (2012). The proportion of cells in the stromal vascular fraction (i.e. total cells after collagenase digestion but before plating, excluding the adipocytes) with pericyte markers is 2.0%, in contrast to the 59.0% of cells with supra-adventitial markers, which do not express SMA (Zimmerlin et al., 2013). The relationship of the supra-adventitial cells to nerves has not yet been investigated.

However, almost 90% of the hADSC were observed closely associated with developing nerves. This suggests at least three hypotheses: that this subpopulation is behaving as perineurial cells (sclerotome-derived); that they were recruited as glial cells, behaving as neural crest cells; or that they adopted a perineural niche as soon as it became available in the embryo.

Under the first hypothesis, hADSC would be recruited by the developing nerves from the adjacent mesenchyme to form the nerve sheaths. Upon the passage of growing axons and establishment of peripheral ganglia, part of the rostral sclerotomal cells interact directly with them and provide this structure with the nerve sheaths (Halata et al., 1990). At E6, the chick perineurium is still forming a loose network of mesenchymal cells surrounding immature Schwann cells (Du Plessis et al., 1996). However, this is one of the least studied of all sclerotomal fates, making it an obstacle to identify early molecules that might have been involved with fate commitment.

The second hypothesis is that part of the cells were not behaving as somite-derived cells, but rather as neural crest-derived cells. In all the stages studied here, part of the hADSC appeared intermingled with migratory NCC and, later, with the peripheral nervous system (Figs 2, 4 and 5). The migration of neural-crest cells is a tightly regulated process that involves various chemokines and signaling pathways (Theveneau and Mayor, 2012), which might be influencing the behavior of hADSC regardless of their embryonic origin. Sympathetic nerve fibers are also involved in regulating the migration of non-neural crest-derived cells in humans, such as primordial germ cells (Mamsen et al., 2012). In this case, the xenograft model can be used to investigate hADSC mobilization and recruitment *in vivo*, an important aspect of cell therapies.

Importantly, a part of the ADSC population could be composed of neural-crest derivatives of the adult. The subcutaneous tissue is composed of multiple cell types, including neural crest-derived cells, such as Schwann cells (Le Douarin and Kalcheim, 1999) and hair-follicle roots (Sieber-Blum et al., 2004). Sowa et al. (2013) investigated the presence of neural crest-derived cells in ADSC obtained from mouse subcutaneous tissue of the trunk, and observed that 1.0–1.5% of hADSC are derived from the neural crest. Here, 5.9% of hADSC in E6 embryos were HNK1, suggesting that they might have adopted glial or sympathoadrenal fates (Fig. 5, supplementary material Fig. S2).

However, several reports have described transdifferentiation of the whole ADSC population into Schwann cells (Chi et al., 2010; di Summa et al., 2013; Kaewkhaw et al., 2011; Radtke et al., 2009; Tomita et al., 2013; Widgerow et al., 2014). Our results suggest a more conservative hypothesis, under which the subpopulation with glial potential might be of neural-crest origin. This possibility has not yet been evaluated in human cells derived from adipose tissue, and will be an important aspect to investigate in future studies.

The third hypothesis addresses the possibility that the majority of hADSC were not inclined toward any differentiation pathway, but instead showed a tropism for the perineural niche. In the bone marrow, non-myelinating Schwann cells of autonomic nerves are an essential component of the quiescent haematopoietic stem cell niche (Yamazaki et al., 2011). Peripheral nerves are in contact with diverse stem cell niches of the adult in addition to the bone marrow, such as the intestinal crypt (Liu et al., 2013), hair follicles (Solanas and Benitah, 2013), and muscle satellite cells (Montarras et al., 2013) niches, as well as being involved in models of regeneration such as salamander limbs and pinna of mouse ears (Kumar and Brockes, 2012). If this hypothesis is correct, the large number of cells observed here in a perineural, quiescent niche would concord with the lack of cells differentiating into bone or cartilage in the embryo. It is an exciting possibility that the interplay between nerves and stromal cells might modulate quiescence and survival in diverse stem cell niches in mammals, including in the adipose tissue, *in vivo*.

### hADSC grafted into presumptive BA1 territory behave similarly to cardiac neural-crest cells

In order to further investigate whether hADSC migrate along neural-crest pathways of the host, hADSC were grafted into the presumptive BA1 region. In the cephalic region, NCC can give rise to most of the head mesenchyme, which in the trunk is composed exclusively of mesodermal derivatives (Le Douarin and Kalcheim, 1999).

The hADSC found at E4.5 in the mandibular bud had the same distribution pattern observed in the trunk, where human cells associated with peripheral nerves and did not disperse through the mesenchyme (Fig. 6). Not all mesenchymal cells grafted into this region migrated in the same way; when we grafted QT6 cells (quail fibrosarcoma) in the same stage and location, they dispersed throughout the mesenchyme and did not associate with nerves (Brito et al., 2008). Interestingly, when quail Schwann cells were grafted into the first branchial arch of the chick, they integrated into facial nerves, differentiated into smooth muscle, fused with skeletal muscles of the face, and did not originate bone or cartilage (Real et al., 2005). In contrast to the grafted hADSC, quail Schwann cells were observed in the HNK1+ portion of the nerve, constituting the glia itself.

However, almost 75% of the hADSC grafted into the cephalic region were found in the outflow tract of the heart. Part of the vagal neural-crest cells migrate into the heart through the arterial arches and colonize the mesenchyme of the truncus arteriosus (Kuo and Erickson, 2011; Waldo et al., 1998). This indicates a wide migratory capacity of the hADSC, which were grafted into the posterior mesencephalic level and migrated similarly to NCC derived from the 4th to 8th rhombomeres of the host (Couly et al., 1996).

### Conclusion

This study proposes that transplantation of human primary cells into chick embryos experiment can be used as a top-down approach, in which a cell population is placed in an instructive environment, allowing one to observe their behavior and then deconstruct it for further study. This system has the advantage of screening diverse properties of a cell population without the prior need for cell markers or presuppositions, and can be adapted to specific questions by changing the stage of the embryo and the grafting site. Thus, this approach is a powerful tool to investigate cell potential, behavior and heterogeneity. Here, this strategy allowed us to provide evidence for a novel cell interaction between hADSC and nerves, raising new questions about their ontogeny, plasticity and niche *in vivo*.

## MATERIALS AND METHODS

### Cells and samples

Primary human skin fibroblasts, human lung carcinoma cell line A549, human breast carcinoma cell line MCF7, and human glioblastoma cell line U87 were obtained from the Cell Bank of Rio de Janeiro (BCRJ, Rio de Janeiro, Brazil).

Fragments of subcutaneous adipose tissue and lipoaspirates were obtained from the abdominal region of 6 surgical patients in the Clementino Fraga Filho University Hospital (HUCFF), Federal University of Rio de Janeiro, Brazil. All the procedures were approved by the Investigational Review Board at HUCFF, and the patients gave their written informed consent.

### Isolation of human adipose tissue-derived stromal cells (hADSC)

hADSC were obtained as previously described (Zuk et al., 2001; Baptista et al., 2009). Briefly, fragments and lipoaspirates were enzymatically digested with 10 mg/ml collagenase IA (Sigma) for 1 h at 37°C under agitation. Cells were plated at 1−2×10^4^ cells/cm^2^ in DMEM (LGC) supplemented with 10% fetal bovine serum (Cultilab) and penicillin/streptomycin (Sigma), and kept overnight at 37°C, 5% CO_2_. Non-adherent cells were removed, and adherent cells were maintained as above and expanded in up to 6 passages.

After *in vitro* expansion, the hADSC used here were homogeneous for the expression of surface antigens described in the literature (including CD105, CD90, CD13 and CD44) and were negative for hematopoietic antigens (including CD45, CD14, CD34, CD3 and CD19), as previously reported by one of the authors (Baptista et al., 2007).

### Cell culture and spheroid formation

Spheroids were prepared as described (Brito et al., 2008). Briefly, cells were seeded at high density (5×10^5^ cells/ml) and plated in 60 mm Petri dishes untreated for cell adhesion (Prolab), using the same culture medium described above, for two days.

### Chicken embryo manipulations

*Gallus gallus* eggs were obtained from Granja Tolomei (Rio de Janeiro, RJ, Brazil) and staged according to HH stages (Hamburger and Hamilton, 1951) or the total number of somites (somite stage, ss). For grafting cells in the somitic region, embryos were incubated at 37°C until at least 13ss. A spheroid of approximately 135 µm in diameter (Fig. 1A) was grafted into the presomitic mesoderm (PSM) of the presumptive 15–20 somites, at the future wing-bud level (Chevallier et al., 1978). The spheroid was inserted into the PSM through a cut in the ectoderm (Fig. 1B). Grafted embryos were reincubated until E3.5, E6 or E8. For grafts in the cephalic region, the embryos were incubated until 5–8ss. Spheroids were grafted in the presumptive first pharyngeal arch as mapped previously (Couly et al., 1996) and the incubation was continued until E4.5 or E6. The conduct of all experiments conformed to the national animal welfare and experimentation guidelines.

### Nile blue sulfate, Alcian blue and haematoxylin-eosin staining

To observe cell death, embryos were washed in PBS (phosphate saline buffer) prior to fixing, incubated for 30 min in a 10 µg/ml Nile blue sulfate solution in PBS, washed for 90 min in PBS, and immediately photographed (Jeffs and Osmond, 1992). For Alcian blue staining, sections were incubated in a solution of 0.5% Alcian blue and 0.5% acetic acid for up to 10 min, followed by incubation in 1% phosphomolybdic acid (PMA) for 10 min (Culling, 1974). Haematoxylin-eosin staining was done according to routine protocols (Culling, 1974).

### Genomic *in situ* hybridization with *Alu* probes

*Alu* probes were synthesized by PCR (Steck et al., 2010). Each reaction contained 1× PCR buffer, 2.0 mM MgCl_2_, 0.1 mM dCTP, 0.1 mM dGTP, 0.1 mM dATP, 0.065 mM dTTP, 0.035 mM dig-11-dUTP (Roche), 0.4 µM of each primer, 2.5 U Taq (Cenbiot) and 50 ng human genomic DNA isolated from ADSC, in a total of 50 µl. The cycling conditions were: initial denaturation of 94°C, 4 min; 40 cycles of 94°C, 20 s, 60°C, 20 s, and 72°C, 40 s; and a final extension of 72°C, 5 min. The primers used were AluFw: 5′-CGAGGCGGGTGGATCATGAGGT-3′ and AluRev: 5′-TTTTTTGAGACGGAGTCTCGC-3′ (Walker et al., 2003).

Embryos were fixed using Formoy solution (Ethanol:Formaldehyde 37%:Acetic acid, 6:3:1), dehydrated with ethanol and xylene, and embedded in Paraplast (Sigma). Serial sections of 7 µm were cut using a manual microtome. Genomic *in situ* hybridization of *Alu* probes on histological sections was performed as previously described (Steck et al., 2010). Probes were localized using anti-digoxigenin-AP, Fab fragments (#11093274910, Roche) in 1:2000 concentration, and were detected using NBT (Roche) and BCIP (Sigma) as substrates. Sections were dehydrated with ethanol/xylene and mounted with Entellan new (Merck).

### RNA *in situ* hybridization and immunostaining

RNA *in situ* hybridization on histological sections was performed as described by Charrier et al. (2002). Chicken-specific antisense RNA probes were synthesized as described previously for *Sox9* (Kordes et al., 2005), *MyoD* (Pourquié et al., 1996) and *Bmp4* (Francis et al., 1994). Immunostaining using mouse monoclonal HNK1/N-CAM/CD57 antibody (Developmental Studies Hybridoma Bank, University of Iowa) was performed as described by Creuzet et al. (2004). Goat anti-mouse IgM-HRP (sc-29743, Santa Cruz) was used as the secondary antibody at 1:50. The alpha-Smooth Muscle Actin 1A4 antibody (M085129, Dako) was used in 1:100 concentration and identified with goat anti-mouse IgG-HRP (G-21040, Novex). Both were revealed using DAB (Sigma).

### Image treatment

Micrographs were taken using an Axioplan microscope, AxiocamHR camera and Axiovision software (Zeiss). Brightness, contrast and color balance of the images were adjusted using Photoshop CS6 (Adobe).

## Supplementary Material

Supplementary information
