# Supplementary material for: Chick embryo xenograft model reveals a novel perineural niche for human adipose-derived stromal cells
Source: Biol Open. 2015 Aug 28;4(9):1180–93. doi: 10.1242/bio.010256 (PMC4582113; doi:10.1242/bio.010256)
Supplement: Supplementary information [file supp_4_9_1180__index.html]

Chick embryo xenograft model reveals a novel perineural niche for human adipose-derived stromal cells — Supplementary information 

# Chick embryo xenograft model reveals a novel perineural niche for human adipose-derived stromal cells

## BIO010256 Supplementary information

**Files in this Data Supplement:**

- Supplementary information
