# Supplementary material for: Chick embryo xenograft model reveals a novel perineural niche for human adipose-derived stromal cells
Source: Biol Open. 2015 Aug 28;4(9):1180–93. doi: 10.1242/bio.010256 (PMC4582113; doi:10.1242/bio.010256)
Supplement: Supplementary information [file supp_bio.010256_BIO010256supp.pdf]

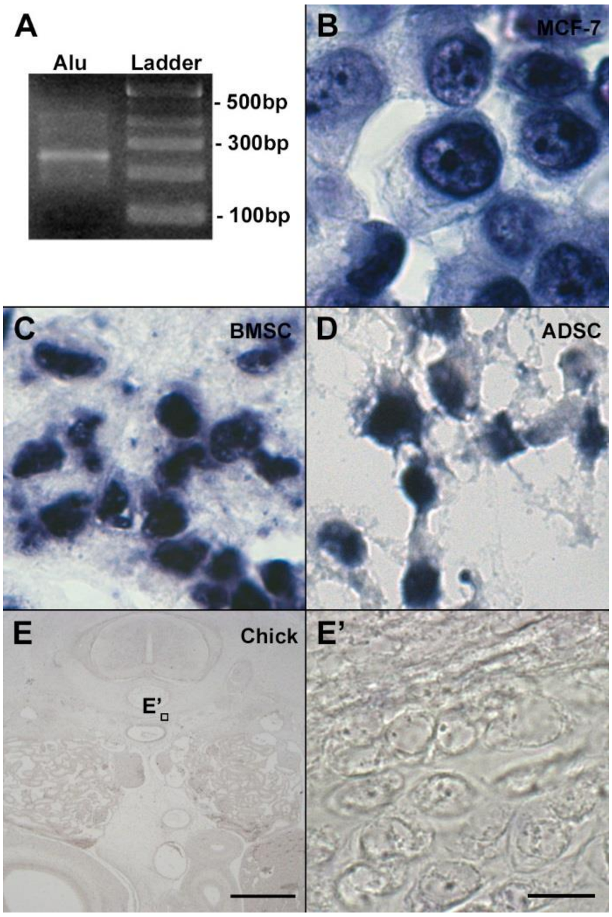

Fig. S1. In situ hybridization with *Alu* probes. (A) Alu DNA probes were synthesized through PCR using digoxigenin-11-dUTP nucleotides. As expected, probes had between 200 and 300 bp. **(B,C,D)** In situ hybridization revealed that *Alu* probes stained only the nuclei of different human samples, illustrated by the tumoral cell line MCF-7 (B), bone marrow-derived stromal cells (C) and adipose-derived stromal cells (D). **(E,E′)** Chick embryos were used as a negative control. No nuclei were found stained. (B,C,D,E′) Scale: 10µm. (E) Scale: 500µm.

Biology Open • Supplementary information

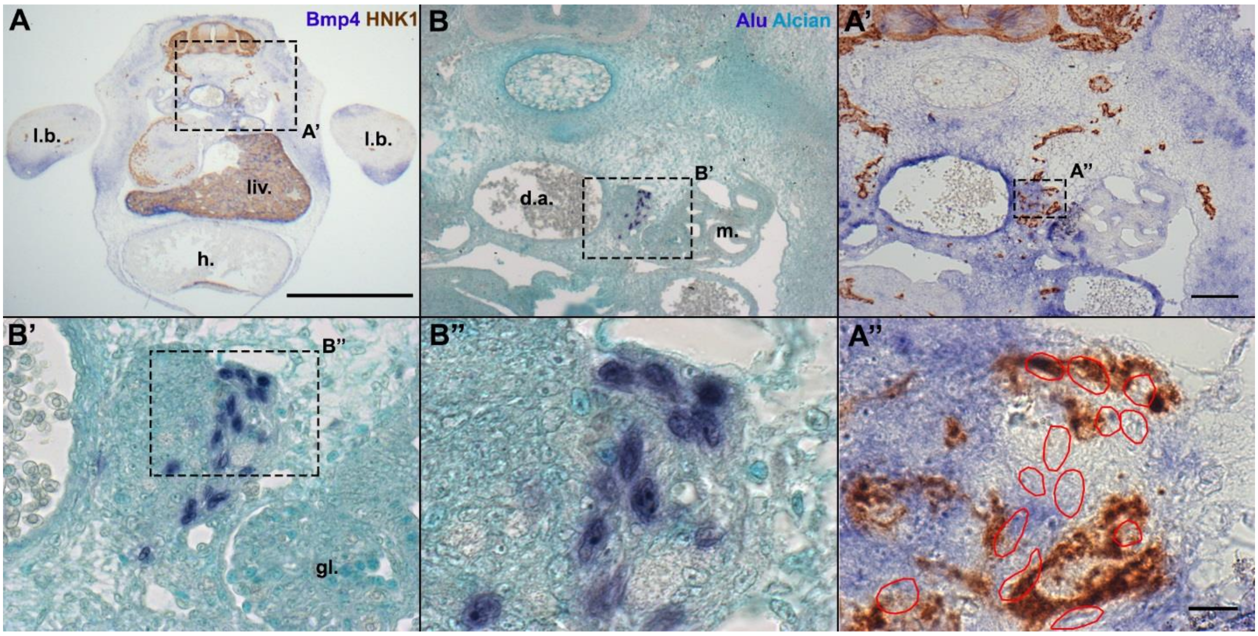

Fig. S2. Some hADSC were observed in the adrenal primordium. (A, A′,A″) In situ hybridization with *Bmp4* RNA probes and immunostaining with HNK1 antibody. Scale: 1mm. **(B,B′,B″)** In situ hybridization with *Alu* probes revealed hADSC in close proximity to the adrenal primordium in one embryo. Counterstaining with Alcian blue. (A″) *Alu*+ nuclei in an adjacent section are indicated with a red outline. hADSC were localized closer to the HNK1+ sympathoadrenal progenitors than to the *Bmp4*+ adrenal cortical cells. (A′,B) Scale: 100µm. (A″,B″) Scale: 10µm. D.a., dorsal aorta; gl., glomerulus; h., heart; l.b., limb bud; liv., liver; m., mesonephros.

Biology Open • Supplementary information

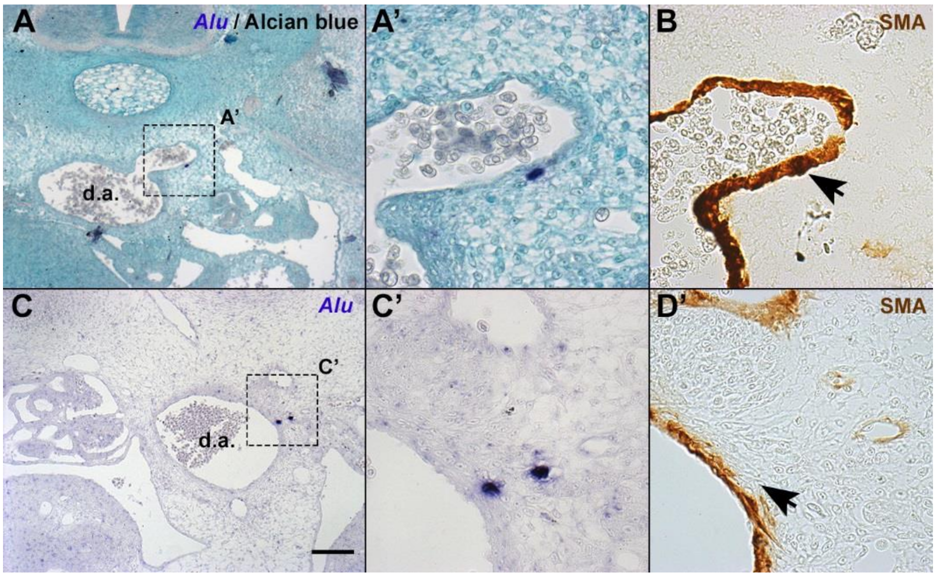

Fig. S3 Perivascular hADSC could be positive or negative for α-Smooth Muscle Actin (SMA). In situ hybridization with *Alu* probes (blue) reveal human cells (black arrows) associated with the dorsal aorta (d.a.). Immunostaining with SMA (brown). **(A,A′,B)** Human cell in a SMA+ perivascular region. **(C, C′, D)** Human cell in na adventitial position, in a SMA- region. Scale: 100µm. (A′,B,C′,D) Scale: 20µm.

Biology Open • Supplementary information

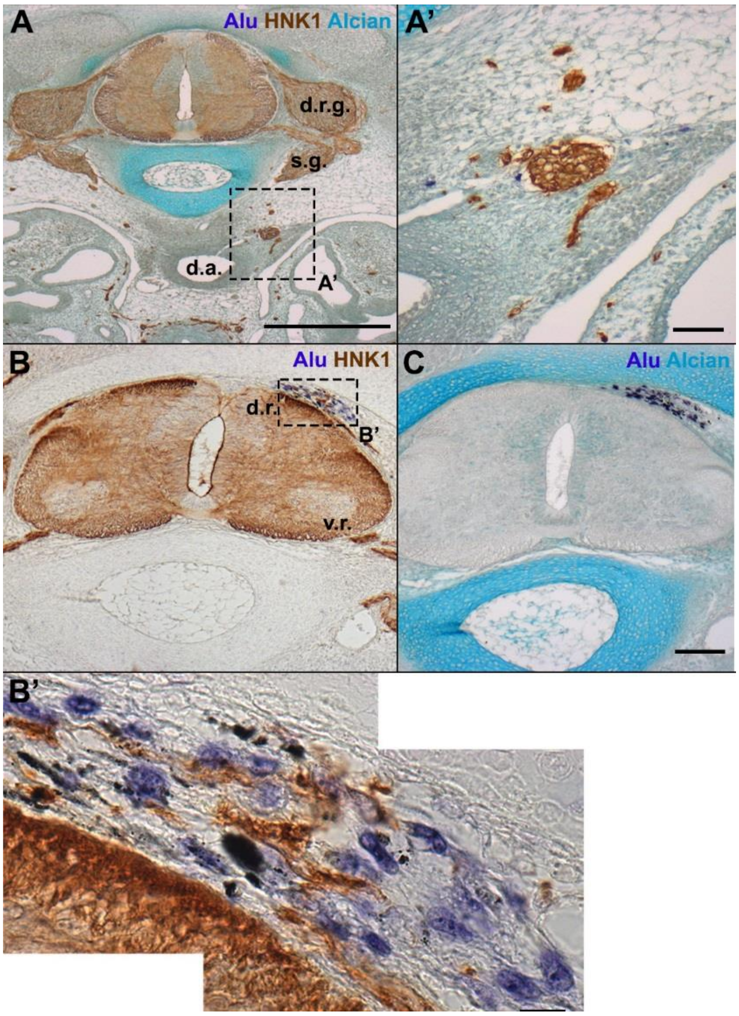

Fig. S4. Distribution of hADSC cells in E8 chick embryos. (A,A′) In situ hybridization with *Alu* probes, immunostaining with HNK1 and cartilage staining with Alcian blue in cross-sections. In one embryo, ASC were localized dorsolateral to the dorsal aorta, close to a HNK1+, ganglion-like structure. (A) Scale: 500µm. **(B,B′)** In another embryo, in situ hybridization with *Alu* probes and immunostaining with HNK1 antibody showed ASC in the region of the dorsal root. (B′) is composed of two partially overlapping micrographs. Scale: 10µm. **(C)** In a serial section to (B), in situ hybridization with *Alu* probes and Alcian blue staining revealed that ASC were not inside the vertebra condensation. (B,C) Scale: 100µm. D.a., dorsal aorta; d.r., dorsal root; d.r.g., dorsal root ganglion; s.g., sympathetic ganglion; v.r., ventral root.

Biology Open • Supplementary information

Table S1. Distribution of human skin fibroblasts cells in E6 chick embryos. The first column represents individual embryos and the respective grafting sites. The other columns represent the number of cells counted in a given location. Cells were manually counted in 7µm sections with intervals of 28µm. Proximity to HNK1+ tissues was identified visually. Sum of *Alu*+ nuclei in a given location, and their percentages relative to total cell count in the embryo, are given in the last row. PSM, presomitic mesoderm.

			Mesenchyme lateral to neural tube and notochord		Mesenchyme ventral to the notochord					
	Dorsal dermis	Adjacent to neural tube	Close to HNK1	Not close to HNK1	Perivascular	Close to HNK1	Perivascular and close to HNK1	Neither	Dorsal aorta (Perivascular)	**Total (per embryo)**
Embryo 1 (15º-16º PSM)	3	5	0	1	12	6	4	18	0	**49**
Embryo 2 (18º-19º PSM)	5	18	48	20	8	4	2	7	6	**118**
Embryo 3 (18º PSM)	3	0	0	3	0	12	1	39	2	**60**
Embryo 4 (18º PSM)	1	0	0	1	0	0	3	3	0	**8**
**Total (per site)**	**12 (5.1%)**	**23 (9,8%)**	**48 (20,4%)**	**25 (10,6%)**	**20 (8,5%)**	**22 (9,4%)**	**10 (4,3%)**	**67 (28,5%)**	**8 (3,4%)**	235

Biology Open • Supplementary information
